# Achieving full-term pregnancy in the vizcacha relies on a reboot of luteal steroidogenesis in mid-gestation (*Lagostomus maximus*, Rodentia)

**DOI:** 10.1371/journal.pone.0271067

**Published:** 2022-07-08

**Authors:** Santiago Andrés Cortasa, Pablo Felipe Ignacio Inserra, Sofía Proietto, María Clara Corso, Alejandro Raúl Schmidt, Alfredo Daniel Vitullo, Verónica Berta Dorfman, Julia Halperin

**Affiliations:** 1 Centro de Estudios Biomédicos Básicos, Aplicados y Desarrollo (CEBBAD) Universidad Maimónides, Buenos Aires, Argentina; 2 Consejo Nacional de Investigaciones Científicas y Técnicas (CONICET), Buenos Aires, Argentina; National Institute of Child Health and Human Development (NICHD), NIH, UNITED STATES

## Abstract

Reactivation of the hypothalamic-pituitary-ovarian (HPO) axis triggered by the decline in serum progesterone in mid-gestation is an uncommon trait that distinguishes the vizcacha from most mammals. Accessory corpora lutea (aCL) developed upon this event have been proposed as guarantors of the restoration of the progesterone levels necessary to mantain gestation. Therefore, the steroidogenic input of primary CL (pCL) vs aCL was evaluated before and after HPO axis-reactivation (BP and AP respectively) and in term pregnancy (TP). Nonpregnant-ovulated females (NP) were considered as the pCL-starting point group. In BP, the ovaries mainly showed pCL, whose LH receptor (LHR), StAR, 3β-HSD, 20α-HSD, and VEGF immunoexpressions were similar or lower than those of NP. In AP, luteal reactivity increased significantly compared to the previous stages, and the pool of aCL developed in this stage represented 20% of the ovarian structures, equaling the percentage of pCL. Both pCL and aCL luteal cells shared similar histological features consistent with secretory activity. Although pCL and aCL showed equivalent labeling intensity for the luteotropic markers, pCL were significantly larger than aCL. Towards TP, both showed structural disorganization and loss of secretory characteristics. No significant DNA fragmentation was detected in luteal cells throughout gestation. Our findings indicate that the LH surge derived from HPO axis-reactivation targets the pCL and boost luteal steroidogenesis and thus progesterone production. Because there are many LHR-expressing antral follicles in BP, they also respond to the LH stimuli and luteinize without extruding the oocyte. These aCL certainly contribute but it is the steroidogenic restart of the pCL that is the main force that restores progesterone levels, ensuring that gestation is carried to term. Most importantly, the results of this work propose luteal steroidogenesis reboot as a key event in the modulation of vizcacha pregnancy and depict yet another distinctive aspect of its reproductive endocrinology.

## Introduction

The corpus luteum (CL) is a transient endocrine structure that plays a central role in the regulation of the estrous cycle, and is required to support uterine development, implantation, and pregnancy. The development of the CL from the remains of the luteinizing hormone (LH)-triggered ovulated follicle marks the onset of the luteal phase of the estrous cycle. During this phase, the CL will produce and release ovarian steroids, mainly progesterone [1]. Mammals display a precise regulation of the steroidogenic pathway to ensure adequate levels of progesterone throughout both non-fecund ovarian cycle and gestation. In addition to the important role of LH in triggering the luteinization of granulosa cells, it is worth highlighting the importance of some LH receptor (LH-R)-downstream targets that are key for progesterone synthesis from cholesterol: steroidogenic acute regulatory protein (StAR), P450 side-chain cleavage (CYP11A1), and 3β-Hydroxysteroid dehydrogenase (3β-HSD) [2, 3]. As the primary transporter of cholesterol from the outer to the inner mitochondrial membrane, StAR is a rate-limiting regulator for steroid production, and thus essential for luteal steroidogenesis [4]. Its activity is upregulated by LH, and in murines by prolactin (PRL) as well [5]. Both LH and PRL were shown to also upregulate 3β-HSD, which converts pregnenolone to progesterone [5–8] whereas PRL represses the expression of 20α-hydroxysteroid dehydrogenase (20α-HSD) which catabolizes progesterone to the biologically inactive 20α-dihydroprogesterone [5, 9]. The success of the steroidogenic pathway in terms of progesterone as the end product of these linked enzymatic reactions depends largely on the availability of the precursor molecule, cholesterol. One of the main potential sources of cholesterol for steroidogenesis comes from the import of cholesterol found in circulating lipoproteins [10]. Therefore, the existence of a capillary network that nourishes the developing CL is vital [11, 12]. As a major initiator of angiogenesis, vascular endothelial growth factor (VEGF) acts by stimulating endothelial cell proliferation and its ovarian expression is induced by LH [13].

Another important aspect that affects the availability of progesterone is related to the mechanisms that control lifespan of the CL, and such varies among mammalian species in both cyclicity and pregnancy. In a recent review, Hennebold has categorized CL in ultrashort-lived CL, short-lived CL, and long-lived CL, according to how long it functions after ovulation in the non-fecund ovarian cycle, and how luteal lifespan is affected by pregnancy onset and gestation [14]. Mice and rats are examples of mammals with ultrashort-lived CLs, which will produce significant amounts of progesterone only if mating, fertilization, or implantation occur. And only implantation leads to a continuous luteal progesterone production and secretion throughout gestation, and precisely on this luteal source relies on the maintenance of pregnancy for these species [5, 15]. On the other hand, primates and domestic farm species have short-lived CL, which develops and functions for a finite interval during the ovarian cycle in the absence of pregnancy, but its function is extended if pregnancy occurs [16, 17]. After implantation, CL continues to produce and secrete progesterone until the placenta becomes steroidogenically active and takes over this function, known as luteal-placental shift [18]. On the contrary, if implantation does not occur, CL rapidly regresses and the decrease in circulating progesterone will remove the negative feedback on the gonadotropin release which will allow a new cycle of follicle maturation and ovulation to occur [1]. Last, for those that have long-lived CL, the lifespan of a fully functioning CL can last for several months for either fertile or non-fertile cycles. Such is the case of dogs, wolves, foxes, cats, ferrets, and skunks [1, 14, 19].

Although within the Order Rodentia, the South American plains vizcacha (*Lagostomus maximus*) display a remarkable long five-month length gestation [20], compared to the very short 20-day length one exhibited by their distant murine cousins. Furthermore, pregnant vizcachas have a biphasic progesterone profile [21] instead of the steady-increase progesterone curve of most pregnant mammals [1]. In addition, early in pregnancy, the onset of placental calcification and thinning of the uterine segmental arteries as they move away from the cervix and toward the ovary strongly suggest that the vizcacha placenta is, at best, steroidogenically deficient [22–24]. As a result of the decrease in circulating progesterone represented in the "valley" of the biphasic curve around day 90 of gestation, and the consequent elimination of negative feedback on gonadotropin release, a new set of accessory CL (aCL) arises in the mid-gestation [21, 25–27]. It has been attributed precisely to these aCL the recovery of the progesterone levels necessary to adequately complete embryonic development and to carry the pregnancy to term [21]. Yet, no luteal regression has been reported throughout the gestation of vizcacha which suggests the persistence of the primary CL (pCL) despite the emergence of the new set of aCL [28, 29].

Given that vizcachas clearly have mechanisms to adjust progesterone levels for a successful gestation that differ greatly from other rodents and mammals in general, in the present work various markers of luteal development and steroidogenesis were analyzed to deepen the understanding of pCL and aCL in terms of progesterone inputs and their role in sustaining pregnancy.

## Materials and methods

### Ethics statement

All experimental protocols performed in the present study were reviewed and authorized by the Institutional Committee on the Use and Care of Experimental Animals (CICUAE) of Universidad Maimónides, Argentina (CICUAE Resolution N° 59/17). Handling and sacrifice of animals were performed in accordance with all local, state, and federal guidelines for the care and utilization of laboratory animals. Husbandry of the animals met the National Institutes of Health Guidelines for the Care and Use of Laboratory Animals [30] and the guidelines of the American Society of Mammalogists (ASM) for the use of wild mammals in research [31]. Appropriate procedures were performed to minimize the number of animals used. The South American plains vizcacha does not constitute an endangered species [32].

### Animals

Twenty-five adult female vizcachas, *Lagostomus maximus*, weighing between 1.9 and 2.5 kg were used throughout the present study. Animals were captured in their habitat using live traps placed at the entrance of their burrows in a natural population site at the *Estación de Cría de Animales Silvestres* (ECAS), Province of Buenos Aires, Argentina (34° 51’ 0” S, 58° 6’ 37” W). The capture and transport of animals were approved by the Ministry of Agriculture Authority of the Buenos Aires Province Government. Animals were housed for one week before euthanasia, under a 12: 12 hour light cycle to simulate their natural luminal exposure (low light of 12 watts followed by moonlight) and 22 ± 2°C room temperature, with ad libitum access to food and tap water.

Animals were grouped according to their reproductive status as the following: pregnant before HPO reactivation (BP), pregnant after HPO reactivation (AP), term-pregnant (TP) and nonpregnant (NP) (Table 1). To establish the different groups, the time of capture was planned according to the natural reproductive cycle of the vizcachas previously described by Llanos and Crespo [33] and based on our own field expertise [34–36]. Pregnant vizcachas were captured during the breeding season that lasts from April to August. Gestational stage was estimated by the time of capture and confirmed during the surgical procedure by the degree of fetal development as previously described [21, 37]. The AP group was set up with pregnant individuals whose ovaries exhibited, at the time of sacrifice, ovulatory stigmata as evidence of either imminent or recent ovulatory event and later confirmed for the presence of aCL (luteinized unruptured follicles) in hematoxylin-eosin-stained ovary sections. NP females were captured in March before the beginning of the reproductive season.

**Table 1:** Characteristics of the reproductive stages.

	Time of capture	Crown-heel length of foetuses (mm)	Ovulatory stigmata	Serum E2 (pg/ml)	Serum P4 (ng/ml)
**NP**	March	-	yes	34.7 ± 8.1	5.71 ± 2.8
**BP**	April	10	no	39.7 ± 4.33	4.96 ± 3.4
**AP**	July	90–115	yes	99.38 ± 14.88	9.98 ± 0.7
**TP**	August	145–156	no	28.56 ± 3.87	0.75 ± 0.43

Two approaches were carried out: 1) the luteal morphology and morphometry were studied in cycling and pregnant animals (NP, BP, AP, and TP), 2) the expression levels of luteotropic markers were studied when the CL develop during the estrous cycle (NP), in pregnancy prior (BP) and post HPO axis reactivation (AP) when accessory CL (aCL) have emerged in the ovary. CL from NP were considered as the starting point of those that will be sustained in pregnancy, and that in this work will be referred as primary CL (pCL). The decrease in progesterone reported in late pregnancy and before parturition indicates a decrease in CL steroidogenesis for this stage [21]. Therefore, ovaries of TP animals were not included in the last approach as the aim was to follow luteal steroidogenesis from the time CL develops during the estrous cycle to the stage of recovery of circulating progesterone levels in mid-pregnancy.

### Animal surgery and tissue sample collection

Animal surgery was performed as previously described in Inserra et al. [34]. Briefly, animals were anesthetized with ketamine chlorhydrate 13.5 mg/kg body weight (Holliday Scott S.A., Buenos Aires, Argentina) and xylazine chlorhydrate 0.6 mg/kg body weight (Laboratorios Richmond, Buenos Aires, Argentina). Blood samples taken by puncture of the inferior vena cava were centrifuged at 3000 rpm for 15 min, and the serum was separated, aliquoted, and stored at -20°C for the subsequent hormonal assays. After bleeding, animals were sacrificed by an intracardiac injection of Euthanyle 0.5 ml/kg body weight (sodium pentobarbital, sodic diphenylhydantoin; Brouwer S.A., Buenos Aires, Argentina). Ovaries were surgically removed and fixed for 48 h in cold 4% paraformaldehyde (PFA) in 0.01 M phosphate-buffered saline (PBS, pH 7.4) for histological and immunohistochemistry studies. For all pregnant females, the number of embryos implanted by uterine horn was recorded and the size of the embryo sacs closest to the cervix was measured with a Vernier caliper.

### Morphological analysis of ovaries

PFA-fixed ovaries of each animal were dehydrated through a graded series of ethanol and embedded in paraffin. Each ovary was serially sectioned at 5 μm thick and mounted onto coated slides. Mounted sections were dewaxed in xylene, rehydrated through a decreasing series of ethanol, and stained with hematoxylin-eosin. Semi-quantification of follicular structures was performed in ovaries of 15 animals (BP: 5, AP: 5, and TP: 5), using ten sections per ovary separated by 300 μm each to avoid counting the same follicle or CL. The criteria adopted for the classification of follicles and CL were based on those proposed by Fraunhoffer et al. [21]. Briefly, all follicles surrounded by one or more layers of cuboidal granulosa cells without an antrum (i.e., primary, secondary, and tertiary) were grouped under the category of ‘preantral follicles’, whereas ‘antral/preovulatory follicles’ were considered when the oocyte was surrounded by multiple layers of granulosa cells, with antrum. Luteinized unruptured follicles (i.e., with a retained oocyte) were classified as aCL while those that did not show a retained oocyte were considered pCL. The primordial follicles represent more than 90% of the total follicular structures of the vizcacha ovary in all reproductive stages [21, 38]. Therefore, to appreciate the differences between preantral, antral and luteal stages throughout pregnancy, the primordial follicles were excluded from the counting. Preantral and antral follicles, and primary and aCL were quantified in the ovaries of vizcachas in BP, AP, and TP to estimate their relative abundance, which was expressed as a percentage of the total count. Counting was performed at 100 X magnification and representative images were captured with an optic microscope (BX40, Olympus Optical Corporation, Tokyo, Japan) fitted with a digital camera (390CU 3.2 Megapixel CCD Camera, Micrometrics, Spain) and the image software Micrometrics SE P4 (Standard Edition Premium 4, Micrometrics, Spain). CL perimeters were delimited and both diameter and average area were determined using Image-Pro Plus software (Image Pro Plus 6, Media Cybernetics Inc, Bethesda Maryland, USA).

### Immunohistochemistry (IH)

To study luteal steroidogenesis, specific antibodies for StAR, 3β-HSD, 20α-HSD, LHR, and VEGF were tested on 5 μm-thick ovary sections in NP, BP and AP (5 animals per group) (Table 2). For each studied marker, three slides (containing 3 tissue sections per slide) corresponding to anterior, middle, and posterior regions of the ovary were tested. Adjacent slides were tested for each marker. Briefly, sections were subjected to antigen retrieval by boiling sections in 10 mM sodium citrate buffer pH 6.0 for 20 min. Endogenous peroxidase activity was quenched, and nonspecific binding sites for immunoglobulins were blocked by incubating sections with 10% normal serum. Immunoreactivity was detected by incubating sections overnight in a humid chamber at 4°C with the primary antibody (Table 2). Immunoreactivity was revealed with a secondary biotinylated goat anti-rabbit IgG or rabbit anti-goat IgG, as appropriated, followed by incubation with avidin-biotin complex (ABC Vectastain Elite kit, Vector Laboratories, Burlingame, USA). Specificity of secondary antibodies was corroborated in adjacent sections by omission of the primary antibodies. The reaction was visualized with 3,3’-diaminobenzidine (DAB kit, Vector Laboratories, Burlingame, USA). Sections were counterstained with hematoxylin or methyl green for morphological orientation, then dehydrated, and mounted. Sections were imaged using an optic BX40, Olympus microscope fitted with a digital 390CU 3.2 Mega Pixel CCD Micrometrics camera.

**Table 2:** List of primary antibodies used.

Antibody	Host Animal	Dilution	Supplier/Source	Catalog number
StAR	mouse polyclonal	1:200	Santa Cruz Biotechnology	sc-166821
3β-HSD	goat polyclonal	1:200	Santa Cruz Biotechnology	sc-330820
20α-HSD	rat polyclonal	1:200	Gibori lab, University of Illinois at Chicago (non-commercial)	
LHR	rabbit polyclonal	1:200	Santa Cruz Biotechnology	sc-25828
VEGF	goat polyclonal	1:200	Santa Cruz Biotechnology	sc-1836

To assess the immunoreactivity levels of each marker, a quantitative analysis was performed determining the immunoreactive area (IRA) using the Image Pro Plus software. Briefly, once the ovarian structures to analyze were selected, those cells that had a gray level darker than a threshold criterion defined as the optic density three times higher than the mean background density were considered for the estimation. The mean background density was measured in a region devoid of specific immunoreactivity, immediately adjacent to the analyzed region. The IRA of a given ovarian structure was determined by measuring the area (𝜇m^2^) covered by threshold pixels (pixels with a gray level higher than the defined threshold density). When it was related to the total area of the ovarian structure, it was expressed as % IRA. The gray level of threshold setting was maintained for all studied sections that were incubated with the same antibody. All images were taken the same day under the same light condition to avoid external variations. Adobe Photoshop CS5 software (Adobe Systems Inc.) was used for digital manipulation of brightness and contrast when preparing the shown images.

### TUNEL assay

Detection of DNA fragmentation/integrity was performed in paraffin-embedded ovary sections of pregnant animals (BP, AP and TP, n = 5 for each group) by terminal deoxynucleotidyl transferase-mediated deoxyuridinetri-phosphate nick-end labeling technique (TUNEL), using the In situ Cell Death Detection Kit (Roche Diagnostics) with fluorescein-tagged nucleotides according to the manufacturer’s protocol. Briefly, tissue sections, deparaffinized as described above, were permeabilized by incubation with 20 μg/ml proteinase K, for 20 min at 37°C in a humid chamber and treated with the TUNEL reaction mixture. Sections were examined in an Olympus BX40 microscope by conventional epifluorescence with UV illumination. For positive control of the TUNEL reaction, tissue sections were incubated with DNase I recombinant (1000 U/ml in 50 mM Tris–HCL, pH 7.5, 10 mM Mg_2_Cl and 1 mg/ml bovine serum albumin) for 10 min at room temperature, whereas sections incubated without the terminal transferase enzyme in TUNEL mixture were used as a negative control. After incubation, slides were thoroughly rinsed and treated again according to the TUNEL protocol. Images were captured with an Olympus Camedia C-5060 camera.

### Enzyme-Linked Immunosorbent Assay (ELISA) for progesterone and estradiol

Serum P4 and estradiol (E2) levels were determined using Progesterone and Estradiol ELISA kits, EIA-1561 and EIA-2593 respectively (DRG Int., Germany) as previously described by our laboratory [39]. Briefly, direct solid phase enzyme immunoassays detecting a range of 0.18–40.0 ng P4/ml and 16–2000 pg E2/ml were developed. Intra- and inter-assay coefficients of variation were 7.0% and 10.5% respectively for progesterone, and 6.5% and 10.0% respectively for estradiol. The absorbance of the solution measured at 450 nm was inversely related to the concentration of progesterone or estradiol in each sample. Calculation of progesterone or estradiol levels was made by reference to the respective calibration curve. All captured vizcachas were evaluated, and their progesterone or estradiol levels are depicted in Table 1.

### Statistical analysis

Data are presented as mean ± standard error of the mean (SEM) and statistical analysis was performed with Prism 6.0 (GraphPad Software Inc., San Diego, California, USA). Normally distributed data were analyzed by Student’s t-test to compare two groups or one-way analysis of variance (ANOVA) with Tukey’s post hoc test was used for multiple comparisons. Differences were considered statistically significant when p <0.05.

## Results

### Follicle counting

**Fig 1:**
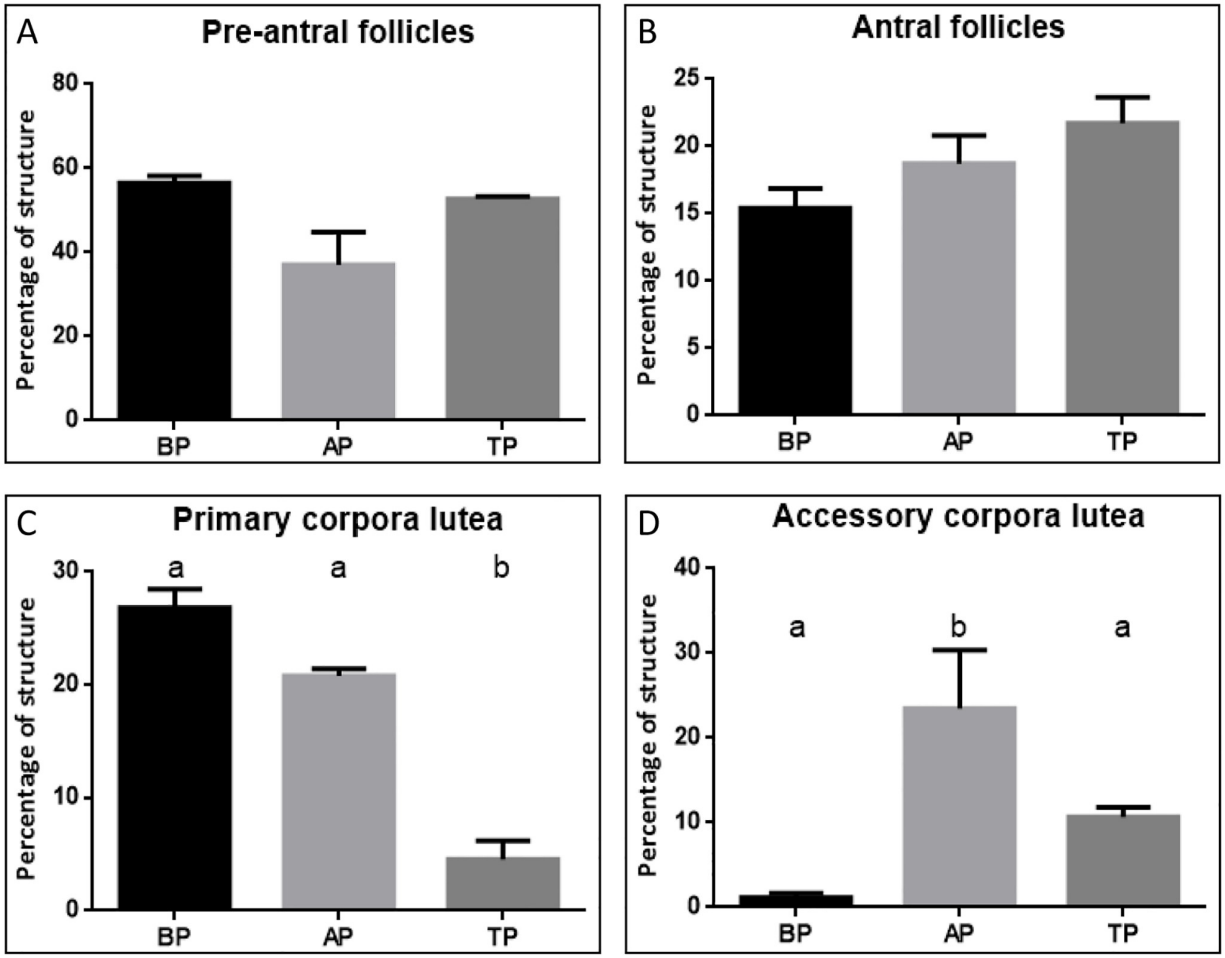
Changes in preantral and antral follicles and corpora lutea populations throughout pregnancy in vizcachas. Preantral and antral follicles, and primary and accessory corpora lutea were quantified in the ovaries of vizcachas before and after HPO axis reactivation (BP and AP respectively), and in term pregnancy (TP) to estimate their relative abundance (n = 5 per stage). Each ovarian structure is plotted as a percentage of the total number of structures counted. Results are expressed as mean value ± SEM. One-way ANOVA test followed by Tukey’s multiple comparisons test: a and b are significantly different among groups (p < 0.05).

Preantral and antral follicles, and pCL and aCL were counted in the ovaries of vizcachas in pregnant animals (BP, AP and TP) to estimate their relative abundance, and were then plotted as percentages of the total structures counted (Fig 1). Preantral follicles were the most abundant, accounting for 40 to 60% of the total follicular structures throughout gestation, highlighting a substantial difference with respect to the rest of the follicular sets (p<0.05, n = 5 per stage) (Fig 1A). Although the percentage of preantral follicles showed some variations throughout gestation, those were not significant. On the other hand, the antral follicles represented approximately 20% of the total follicular structures in the ovaries of the pregnant vizcachas, and this percentage did not vary significantly among the stages of pregnancy (Fig 1B). The most significant result of the count was revealed by the post hoc test and pointed to the marked increase in the amount of aCL in ovaries of AP animals. The aCL went from having a very low presence to none in ovaries of BP animals to represent more than 20% of the total accounted structures in AP, equaling the percentage of pCL recorded in this gestational stage (Fig 1C). Last, both pCL and aCL significantly decreased their abundance in ovaries of TP females compared to those of the previous stage (Fig 1C and 1D).

### Histological study of the corpora lutea

**Fig 2:**
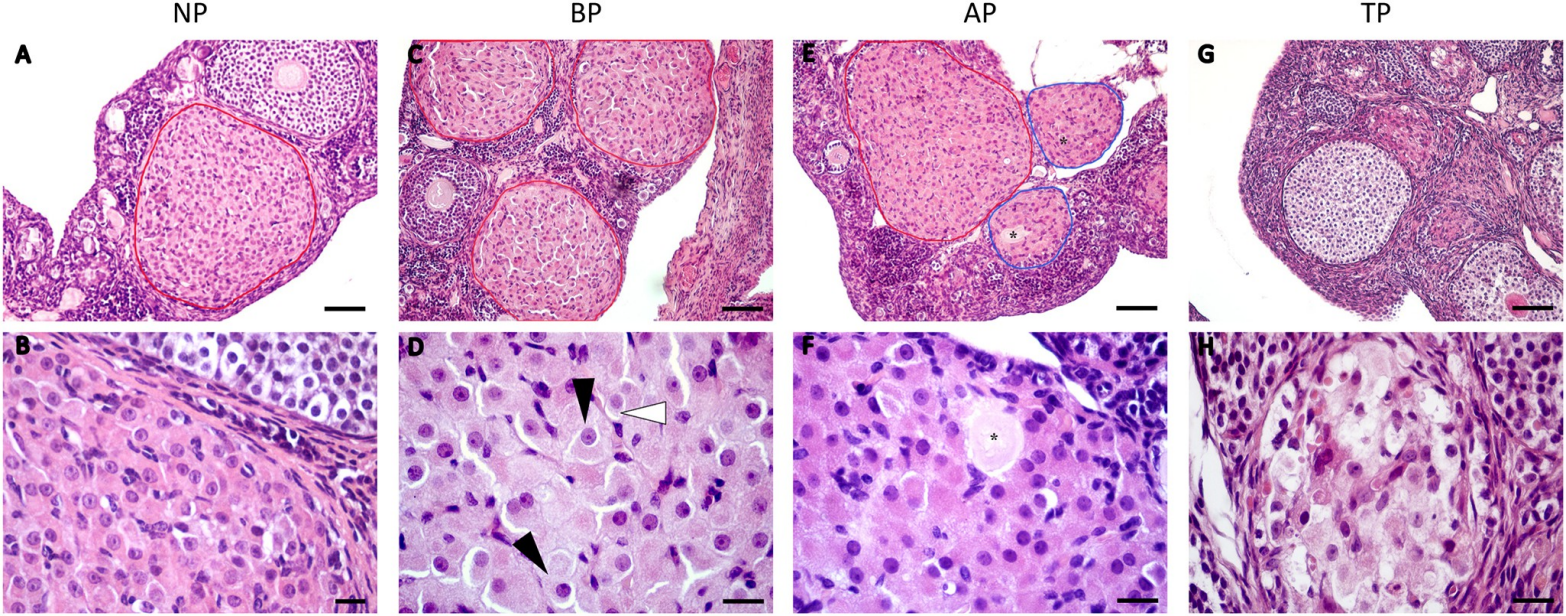
Follow-up of the corpora lutea during the gestation of the vizcacha. Photomicrographs of hematoxylin-eosin-stained ovarian sections depict morphologic and histologic features of corpora lutea throughout pregnancy. Before and after HPO axis reactivation in mid-pregnancy (BP and AP respectively), term pregnancy (TP). Primary corpora lutea outlined in red. Accessory corpora outlined in blue. Black arrowheads indicate large luteal cells and white arrowheads indicate small luteal cells. * indicate a retained oocyte. Scale bar for upper panel: 100 μm. Scale bar for lower panel: 30 μm.

The histological characteristics of corpora lutea were studied in cycling (NP) and pregnant animals (BP, AP and TP) (Fig 2). The ovaries of NP, BP and AP groups did not show major differences regarding the luteal cell histology and overall pCL structure (Fig 2A, 2C and 2E, outlined in red). In these three groups, which emulate the natural progression of pCL from their development upon ovulation during the estrous cycle to the post HPO axis reactivation during pregnancy, it was observed that the cellular composition of the pCL was mainly given by populations of small and large luteal cells (Fig 2B and 2D). Large luteal cells were markedly predominant over the small ones and showed centralized circular nuclei with a differentiated nucleolus and a large cytoplasm with abundant granules. Compacted between the large cells, scarce small luteal cells showed nuclei with slightly more condensed chromatin and less colored cytoplasm (Fig 2B and 2D). Small vessel endothelial cell nuclei were also observed. On the other hand, the appearance of a set of aCL (outlined in blue) was observed in the ovaries of AP (Fig 2E). All aCL showed trapped oocytes (Fig 2E and 2F) and they were noticeably smaller than pCL. The trapped oocytes were observed with different degrees of deterioration in the aCLs. Regardless, the luteal cells of the aCLs appeared healthy and functional, showing similar histological characteristics to those of the pCLs (Fig 2F).

**Fig 3:**
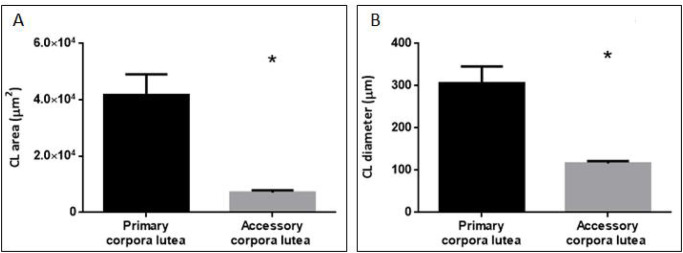
Average size of the primary corpora lutea (pCL) vs accessory corpora lutea (aCL). Panel A: size expressed as mean luteal area ± SEM. Paired sample t-test (p <0.001). Panel B: size expressed as mean diameter ± SEM. Paired sample t-test (p <0.05). *Indicates significant differences.

The average pCL diameter remained relatively even from the time they developed after ovulation (NP) to AP (306.03 ± 39.91 μm). In AP, when coexistence of healthy and functional pCL and aCL were observed in the ovary, the average area of pCL tripled that of aCL (p<0.001; Fig 3A) and such significant difference was also recorded in the average diameter between both structures (p<0.05; Fig 3B). Towards term pregnancy, a structural disorganization of all CLs was observed, characterized by invasion of interstitial tissue and vacuolization in luteal cells. Such disorganization challenged the task of differentiating between pCL and aCL. Likewise, a notable decrease in the size of the CLs was observed at this stage (Fig 2G and 2H).

### DNA integrity assay in luteal cells

**Fig 4:**
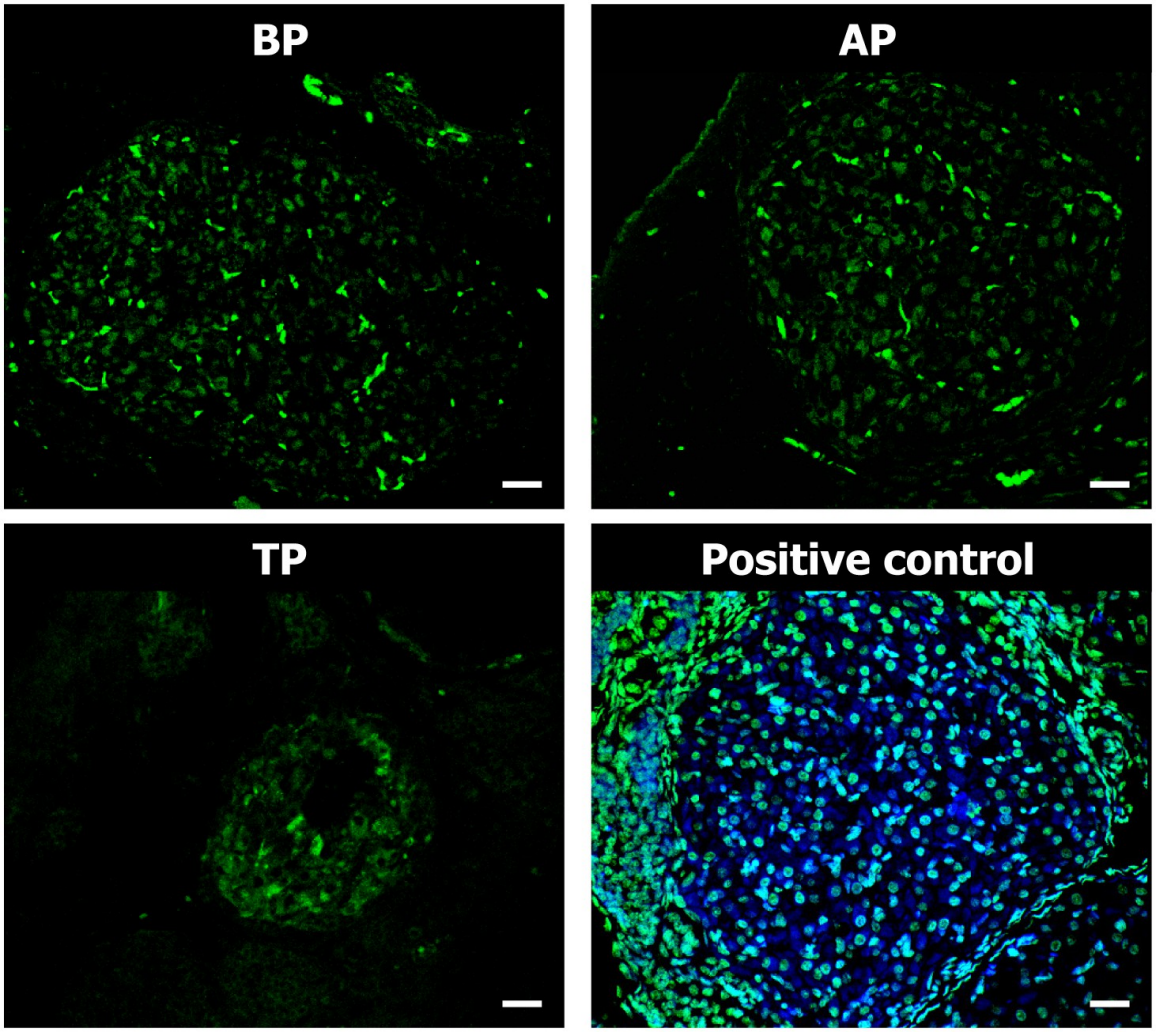
TUNEL (terminal deoxynucleotidyl transferase-mediated deoxyuridinetri-phosphate nick-end labeling) assay for detection of DNA fragmentation of apoptotic luteal cells during vizcacha pregnancy. Confocal laser imaging of TUNEL (green)-stained ovarian sections of females before and after HPO axis reactivation in mid-pregnancy (BP and AP respectively), and in term pregnancy (TP). White arrowheads point to autofluorescent endothelial cells. Positive control double stained with TUNEL (green)-DAPI (blue). Scale bar: 50μm.

To evaluate DNA fragmentation as an indicator of the last phase of apoptosis, sections of ovaries at the three stages of pregnancy were studied by TUNEL assay (Fig 4). Minor signs of luteal apoptosis were observed during gestation, even in TP, when the disorganization of the luteal structure was notorious. Notwithstanding negligible compared to the real DNA fragmentation of the positive control, the red blood cells present in the CL of BP and AP animals showed autofluorescent signal. Yet, such signal almost disappeared in either pCL or aCL in ovaries of TP animals (Fig 4).

### Expression of luteal markers

**Fig 5:**
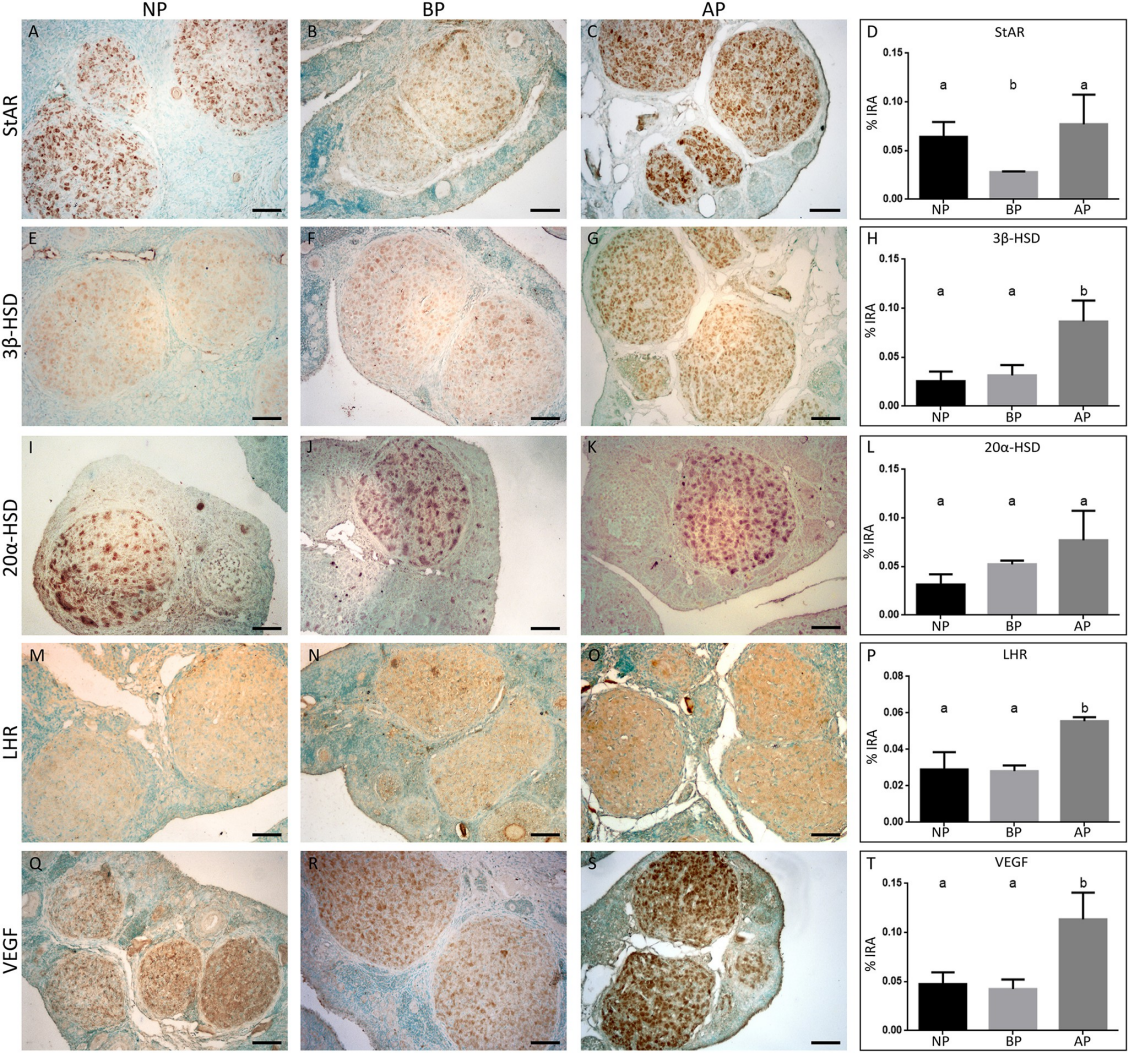
Immunohistochemistry for steroidogenic markers in ovarian sections of cycling and pregnant vizcachas. Photomicrographs of ovarian sections of nonpregnant females (NP) and females before and after HPO axis reactivation in mid-pregnancy (BP and AP respectively) (n = 5 per stage). Sections were immunostained for StAR (A-C), 3β-HSD (E-G), 20α-HSD (I-K), LHR (M-O), and VEGF (Q-S). Brown color indicates positive staining. Methyl-green used as counterstaining. Scale bar: 50 μm. Expression levels were plotted as a percentage of the immunoreactive luteal area (% IRA, last panel). One-way ANOVA test followed by Tukey’s multiple comparisons test: a and b are significantly different among groups (p <0.05).

Luteal expression of steroidogenesis markers was investigated by immunostaining using antibodies reacting with StAR protein and 3β-HSD and 20α-HSD enzymes, LHR and VEGF (Fig 5). The three proteins related to progesterone synthesis, StAR protein and 3β-HSD and 20α-HSD enzymes, were abundantly expressed in the cytoplasm of luteal cells in the three stages analyzed, although the percentage of the immunoreactive luteal area (% IRA) of each marker showed variations between stages. In BP, the % IRA for StAR values decreased significantly compared to that of the NP group (Fig 5A–5D). Then, in AP, the values recovered and largely reached those recorded during NP. For 3β-HSD, the % IRA was relatively similar between the NP and the BP group, but it almost tripled in the corpora lutea of AP animals (Fig 5E–5H). 20α-HSD, which catabolizes and inactivates progesterone, showed similar % IRA along the three analyzed stages (Fig 5I–5L).

The upstream modulator of steroidogenic enzymes, the LHR, also showed significantly higher % IRA in AP, compared to earlier stages (Fig 5M–5P). The expression of this marker was also evidenced in antral follicle granulosa cells in ovaries of pregnant animals (Fig 5N). Last, the luteal activity marker related to the induction of angiogenesis, VEGF, was also evaluated. Similar to that observed for the 3β-HSD enzyme, the % IRA of VEGF did not vary between NP and BP females, but it doubled in AP (Fig 5Q–5T).

**Fig 6:**
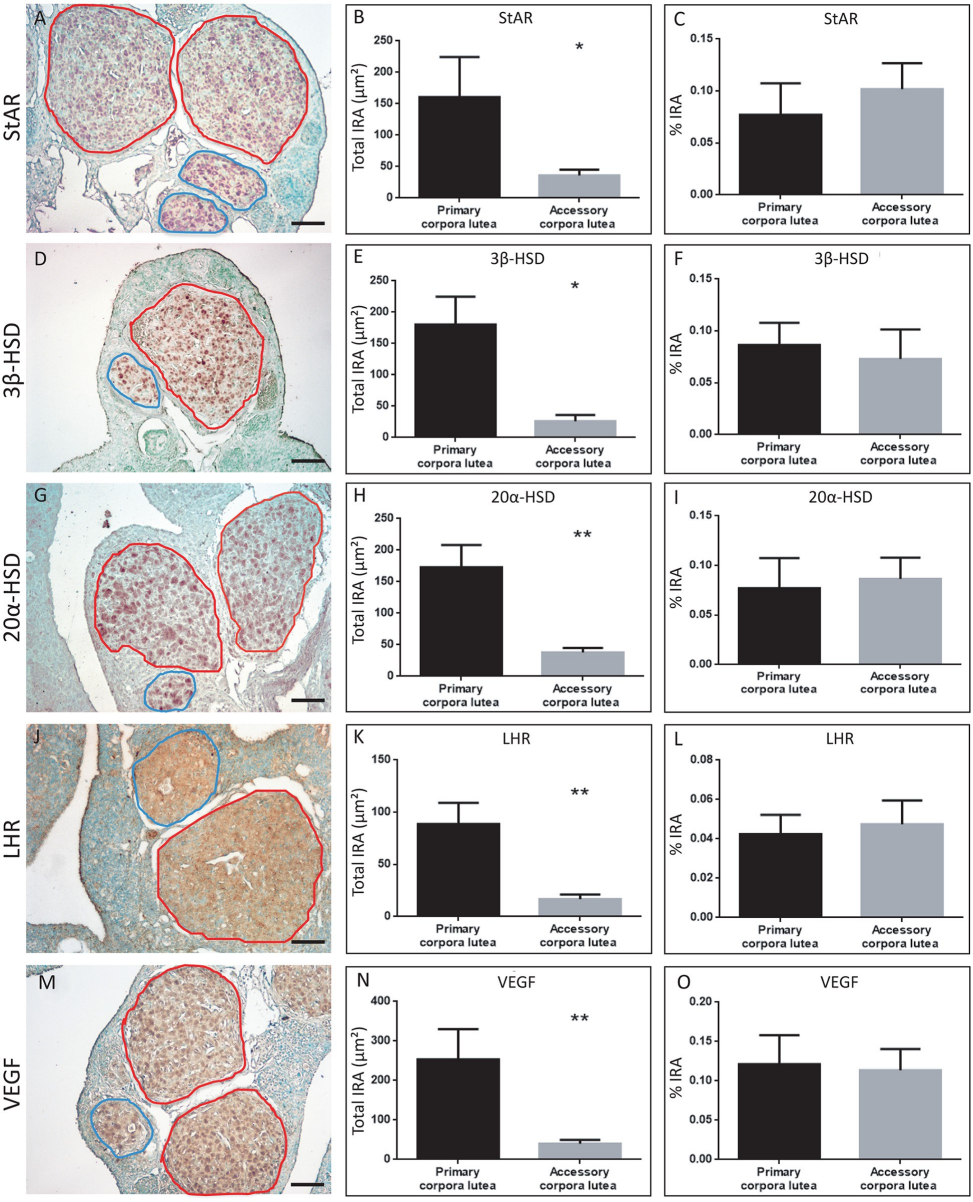
Estimation of steroidogenic input of primary CL vs accessory CL. Left panel: photomicrographs of ovarian sections after HPO axis reactivation in mid-pregnancy immunostained for StAR (A), 3β-HSD (C), 20α-HSD (E), LHR (G), and VEGF (I). Primary corpora lutea surrounded in red and accessory corpora lutea in blue. Brown color indicates positive staining. Methyl-green used as counterstaining. Scale bar 50 μm. Middle panel: measurement of the total immunostained area (Total IRA) of primary versus accessory corpora lutea (B, E, H, K, and N). Right panel: relative measurement of immunostained area (% IRA) of primary versus accessory corpora lutea (C, F, I, L, and O). Data is expressed as mean ± SEM. The significance of the differences was calculated by a Student’s t-test, in which * p <0.05 and ** p <0.01.

The aCLs that arose in the AP showed positive reactivity with all the analyzed markers and exhibited a marking intensity equivalent to that observed in pCL (Fig 6C, 6F, 6I, 6L and 6O). Therefore, and to distinguish the contribution of each type of luteal structure per marker, the average areas of pCL and aCL were calculated for each immunohistochemical assay (Fig 6). For each one of the five markers analyzed, the total IRA in the pCL that was at least four times higher than that calculated in the aCL (Fig 6B, 6E, 6H, 6K and 6N).

## Discussion

Plains vizcachas (*Lagostomus maximus*) are South American wild rodents that belong to the Hystricognathi infraorder and stand out for having certain distinctive aspects of their reproductive physiology. Some of these have been early described by Barbara Weir [20, 40] and are related to their long pregnancies (around 155 days), long estrous cycles, very low litters of well-developed newborns, and an extraordinarily high ovulation rate.

Almost a decade ago, our laboratory reliably established reactivation of hypothalamic GnRH and pituitary LH pulsatilities well into the gestation of vizcachas [25, 26]. This feature is related to a conspicuous characteristic exhibited by the ovaries of *L*. *maximus* (Family Chinchillidae) and shared by other hystricomorphs as the African mole-rats (Family Bathyergidae), and Old-World porcupines (Family Hystricidae): the existence of luteinized unruptured follicles (i.e., accessory corpora lutea, aCL) [41–44]. Although at that time the hypothalamic reactivating mechanism inducing aCL formation was yet unknown, Weir postulated for those aCL a role in supporting the prolonged gestation of these species. Subsequently, it was established that the circulating progesterone of pregnant vizcachas exhibits a biphasic behavior where the hormonal upturn begins after reaching a trough threshold around day 90 of gestation, and such progesterone recovery is in tune with the appearance of the aCL that persist until late pregnancy, advocating Weir’s proposition [21]. Yet, the primary corpora lutea (pCL), whose declining progesterone production would trigger the reactivation of GnRH and LH pulsatility, persist until term as well, without signs of regression by apoptosis [28, and confirmed in the present work].

Our data suggest that the reactivation of the HPO axis in mid-pregnant vizcachas goes far beyond the generation of a new set of aCL. The striking outcome was unveiled by the steroidogenic resumption of the pCLs. Immunoexpression profiles of StAR, 3β-HSD, and VEGF indicate that those ovarian structures that express LHR and, therefore, can respond to the luteotropic stimulation derived from the reactivation of GnRH pulsatility, turn on steroidogenesis and angiogenesis. This naturally affects the LHR-expressing antral follicles, and although they do not go as far as to extrude the oocyte, they do luteinize and develop as steroidogenically actives aCLs. Our morphometric determinations indicate that even though aCL equaled the amount of pCL during mid-pregnancy, the former represent approximately a third of the area of the latter. Therefore, at equivalent staining intensities of the luteal markers and similar histological characteristics of both types of CL, it is reasonable to assume that the upturn progesterone production at mid-pregnancy is mainly driven by the pCL. But beyond establishing which is the luteal structure that wins the progesterone-restoration challenge, one must not lose sight of the fact that a very unusual mechanism, such as the resumption of luteal steroidogenesis, occurs in the midst of gestation in the vizcacha.

For many other rodents, mainly mice and rats, an embryonic diapause ensues when conditions are not optimal to guarantee the best chances of survival of the offspring after birth [45, 46]. The relatively lesser concentration of progesterone-derived from a stressor input is the necessary condition for induction of embryonic diapause in these species. Then, after the adequate stimulus, the inactive corpora lutea can reactivate causing an increase in plasma steroid levels and the resumption of blastocyst growth and development [46–49]. While there is a certain parallel in the luteal steroidogenic fate between these species in diapause and the vizcachas, there is a major difference as well. Quiescence of the CL is facultative for the former (it depends on whether or not there is a stressor) [46] while in the latter, it is part of the physiological modulation of the reproductive axis.

Based on how long CL functions after ovulation in the estrous cycle and how luteal lifespan is affected by pregnancy onset and gestation, mice and rats have been classified as species of ultrashort-lived corpora lutea [14, 50]. They do not develop functional corpora lutea unless pseudopregnancy or pregnancy have ensued, and in the latter case, corpora lutea do not regress to be superseded by another steroidogenic structure during pregnancy, i.e., luteal-placental shift [18]. So, maintenance of the short 20 day-length gestation depends on the continuous production and secretion of luteal progesterone [5]. Similarly, pCL of the vizcachas does not regress after emergence of the aCL at mid-pregnancy and although it has not yet been conclusively proven, early calcification of the placenta and a limited vascular development pose it as a steroidogenically deficient organ [22, 38]. Therefore, maintenance of myometrial quiescence throughout pregnancy of vizcachas would also rely on the production of luteal progesterone. Still, it would be inaccurate to place vizcachas with the ultrashort-lived corpora lutea species. Although the life expectancy of the corpora lutea during the 45 day-length of vizcachas estrous cycle is, to our knowledge, yet uncertain, our results show that CL of nonpregnant have secretory cells with a significant expression of luteotropic markers, compatible with the steroid levels recorded at this stage [21, and this work]. Hence, it can be assumed that nonpregnant vizcachas have steroidogenically active CL, in contrast to the CL of the murine non-fertile cycles which are ephemeral and produce negligible amounts of steroids or even nothing [14, 50].

Other differences in the reproductive endocrinology separate vizcachas from murids with ultrashort-lived corpora lutea. Polyovulation is a common trait for rodents in general, but it is remarkably high for hystricomorphs and particularly for the plains vizcacha, it can reach up to 800 ovulated oocytes being one of the highest rates so far recorded among mammals [40, 41, 51]. Conversely, out of the 10 to 12 implanted embryos, only 1 or 2 successfully complete development and are born well developed (eyes open, teeth, and body fur) [38, 40, 52, 53] compared to the litters of mice and rats that can easily reach twelve comparatively less developed pups that heavily rely on mother’s care for survival [54].

Nor would it be appropriate to place the vizcacha among the short-lived corpus luteum species. Vizcachas share with these the development of a functional corpus luteum after ovulation that lasts a window of time sufficient to allow the embryo to travel through the oviduct and to prepare the uterus for implantation [28, 51]. Yet, if pregnancy ensues in short-lived corpus luteum species, the CL will either last until undergoing luteal-placental shift (primates and some ungulates) [18, 55, 56] or will persist and sustained luteal progesterone production for the whole gestational process (pigs and cows) [50, 57]. For vizcachas, the present results indicate that although the CL show expression of steroidogenic enzymes throughout pregnancy, they do not do so in a sustained manner but with a marked decrease in BP followed by a recovery in AP.

In summary, three known pathways could lead to a process of decline of luteal steroidogenesis in pregnant mammals: 1) diapause [45]; 2) luteal-placental shift [18], and 3) imminence of labor [58]. In the case of the vizcacha, there is no evidence of any sort of embryonic development arrest during early gestation and that it resumes later on [37, 59]. There are also no reports on significant placental steroid production that would enable a luteal-placental shift. Last, it is unlikely that the decline in progesterone levels in early pregnancy acts as a triggering signal for parturition since the trough of the progesterone curve occurs almost three months prior labor [21].

Progesterone is key to successfully maintaining pregnancy in mammals and the corpus luteum as a source of this hormone is essential, at least in the early stages of pregnancy. Clearly, the regulation of luteal steroidogenesis during pregnancy of the plains vizcacha cannot be explained solely for one of the reasons mentioned above. Therefore, it may be necessary to consider other elements to comprehend the mechanism selected by this species to ensure adequate amounts of progesterone and maximize the chances of survival of the offspring. Perhaps to better understand the biological significance of luteal steroidogenic rebooting during pregnancy in vizcachas, future research should focus on evaluating its relationship with the natural sequential embryonic reabsorption that begins early in gestation [23, 24].

## Supporting information

S1 Data (PZFX)

S2 Data (PZFX)

S3 Data (PZFX)

S4 Data (PZFX)
